# The Azeri Version of European Organization for Research and Treatment of Cancer Core Quality of Life Questionnaire (EORTC QLQ-C30): Translation and Validation

**DOI:** 10.31557/APJCP.2020.21.1.267

**Published:** 2020

**Authors:** Mahammad M Davudov, Iraj Harirchi, Namig Amiraliyev, Elnara Mehtiyeva, Zoheir Mirzajani, Kanan Amiraliyev, Narmin Rustamli, Jayran Zebardast, Ali Montazeri

**Affiliations:** 1 *Cancer Research Center of Cancer Institute, Tehran University of Medical Sciences, *; 4 *Cognitive Science Special Linguistics, Institute of Cognitive Sciences, *; 5 *Population Health Research Group, Health Metrics Research Center, Iranian Institute for Health Sciences Research, *; 6 *Faculty of Humanity Sciences, University of Science and Culture, ACECR, Tehran, Iran, *; 2 *Department of Oral and Maxillofacial Surgery, *; 3 *Department of Oncology, Azerbaijan Medical University, Baku, Azerbaijan. *

**Keywords:** Quality of Life, EORTC QLQ-C30, Azerbaijan

## Abstract

**Objective:**

Quality of life in cancer patients has become an important outcome measure. This study aimed to translate and validate the European Cancer Research and Treatment Core Quality of Life Questionnaire (EORTC QLQ-C30) in Azerbaijan.

**Methods:**

Forward-backward procedure was applied to translate the EORTC QLQ-C30 from English into Azeri. Then a cross sectional study was conducted to validate the questionnaire in Azerbaijan. A sample of patients with confirmed diagnosis of oral cancer completed the Azeri version of the questionnaire from January 2017 to December 2018. Construct validity was assessed by performing know groups comparison and item-scale correlation matrix. Reliability was examined by estimating the Cronbach’s alpha coefficient for internal consistency.

**Results:**

In all 141 patients with oral cancer participated in the study. Known groups comparison indicated that the Azeri version of EORTC QLQ-C30 well differentiated between patients who differed in the disease stage. Those with higher stage reported lower functioning and higher symptoms. In addition item-scale correlation matrix showed a good correlation between items and its own hypothesized subscales as expected (Pearson correlation coefficient ranging from 0.735-0.978). The Cronbach’s alpha coefficient ranged form 0.68 to 0.94 indicating acceptable results for the internal consistency of the questionnaire.

**Conclusion:**

This preliminary validation study proved that the Azeri version of EORTC QLQ-C30 is a valid measure of quality of life in cancer patients. However, studies with other cancer patients and stronger psychometric evaluations are recommended.

## Introduction

Cancer and side effects of its treatment are often associated with decrease in quality of life (QoL) (Shapiro, 2018). Although progress in cancer treatment improved outcomes, the disease still has a major impact on patients’ physical and psychological conditions (Jitender, et al., 2018). This may also affect patients’ social activities, and mental health (Naughton and Weaver, 2014). Above all it is argued that even if the tumor completely treated, quality of life of cancer patients would be disturbed to a great deal (Strayhorn et al., 2019). This is especially true for some cancers such as spinal cord, brain stem, salivary glands, orbit, inner ear and jaw and mouth origin (Gegechkori et al., 2017; Davudov et al., 2019; Jehn et al., 2019). Therefore, a careful analysis of the effect of treatment on quality of life in these patients is very important. Indeed the European Organization for Research and Treatment of Cancer (EORTC) has provided a questionnaire namely the EORTC QLQ-C30 to assess the effects of illness and treatment on everyday life of cancer patients (Aaronson et al., 1993). The EORTC QLQ-C30 has been used in over 3,000 studies as a core questionnaire to measure quality of life among cancer patients. The questionnaire has been translated and approved in over 81 languages (https://qol.eortc.org) and currently it is available in Persian (Montazeri et al., 1999), Turkish (Guzelant et al., 2004), Arabic (Awad et al., 2008), Korean (Yun et al., 2004), Japanese (Kobayashi et al., 1998), Chinese (Zhao and Kanda, 2000; Wan et al., 2008), and Spanish (Arraras et al., 2002), just to name a few. However, at present the Azeri version of the EORTC QLQ-C30 is not available. Thus we aimed to translate and validate the questionnaire in Azerbaijan.

## Materials and Methods

*The questionnaire*

The European Organization for Research and Treatment of Cancer Quality of Life Questionnaire (EORTC QLQ-C30) is a core quality of life measure for cancer patients. It contains 30 items and consists of 5 functioning subscales (physical, role, emotional, cognitive and social functioning), one global quality of life subscale, and a number of symptom subscales such as fatigue, pain, and nausea and vomiting. Scores on functioning and global quality of life subscales range from 0 to 100 where the higher scores indicate better conditions. Scores on symptom subscales also range from 0 to 100 but the higher scores indicate greater symptoms (Aaronson et al., 1993, Fayers et al., 2001).

*Translation*

First permission was asked from the EORTC Study Group on Quality of Life. Then forward-backward translation procedure was applied to translate the English-language version of the questionnaire into Azeri as recommended (Cull et al., 2002). As such two independent experts translated the questionnaire into Azeri. Consequently after reviewing both translations a single Azeri version was provided. Then, two other bilingual physicians not connected to the study back translated the questionnaire into English. Subsequently a single English version was provided and checked with original questionnaire. Finally the provisional Azeri version of the questionnaire was pre-tested and its final form was approved by the EORTC Study Group on Quality of Life and was administered in this study.

*Validation*

A cross section study was conducted on a sample of Azeri patients with confirmed diagnosis of oral cancer attending to a teaching hospital affiliated to Azerbaijan Medical University in Baku, Azerbaijan from January 2017 to December 2018. All patients were candidate for surgery. There were no restrictions for including patients in the study with regard to age, gender and disease stage unless they did not wish to participate. Patients completed the Azeri version of the EORTC QLQ-C30 and then the following procedures were applied:

Construct validity: it was assessed using known groups comparison where the disease stage was utilized as known groups indicator. In addition we performed item-scale correlation matrix. As such we hypothesized that items belonging to a subscale should have higher correlation with own subscale.

Reliability: internal consistency was estimated in order to examine reliability.

*Statistical analysis*

Descriptive statistics was used to explore the data. Since the quality of life data was not distributed normally, Kurskal-Wallis test was used for known groups comparison. The Spearman correlation coefficients were estimated in order to provide item-scale correlation matrix. Reliability was estimated using the Cronbach’s alpha coefficient. The alpha value of 0.70 and above was considered as acceptable.

## Results

*Patients*

In all 141 patients with confirmed diagnosis of oral cancer were entered into the study and completed the Azeri version of the EORTC QLQ-C30. The mean completion time for the questionnaire was 9.5 (SD = 1.31) minutes ranging from 8 to 15 minutes. The mean age of patients was 59.5 (SD = 10.7) years. Most patients were male (n = 111, 78.7%) and had stage I of the disease (n = 59, 41.8%). The characteristics of patients are shown in Table 1. 

*Construct validity*

The results obtained from known groups comparison are presented in Table 2. As expected the questionnaire well differentiated between patients who differed in the disease stage. Those with higher stage of the disease scored lower on functioning subscales and scored higher on symptom subscales. In addition performing item-scale correlation analysis the results found to be satisfactory. There were higher correlation between items and its own hypothesized subscale as expected. The findings are shown in Table 3.

*Reliability*

The internal consistency of the questionnaire as assed by the Cronbach’s alpha coefficient ranged from 0.68 to 0.94 indicating acceptable results. The results are presented in Table 4 where descriptive statistics for functioning and symptom subscales also are shown.

**Table 1:** The Characteristics of Study Samples (n = 141)

	No. (%)
Gender	
Male	111 (78.7)
Female	30 (21.3)
Stage	
I	59 (41.8)
II	50 (35.5)
III	21 (14.8)
IV	11 (7.9)
Pre-surgical adjuvant therapy	
Yes	73 (51.8)
No	68 (48.2)

**Table 2:** Quality of Life among the Study Samples by Stage of the Disease as Measured by the Azeri Version EORTC QLQ-C30 by Stage (Known Groups Comparison)

	Stage 1	Stage 2	Stage 3	Stage 4	P*
	Mean (SD)	Mean (SD)	Mean (SD)	Mean (SD)	
Functioning**					
Physical	98.5 (2.7)	83.7 (8.7)	53.9 (6.6)	29.5 (12.1)	< 0.001
Role	87.5 (15.1)	73.3 (17.8)	59.5 (17.9)	33.3 (19.2)	< 0.001
Emotional	74.1 (14.5)	68.1 (18.6)	46.8 (27.3)	41.6 (22.1)	< 0.001
Cognitive	90.1 (15.8)	64.2 (24.8)	23.8 (13.1)	80.2 (25.1)	< 0.001
Social	82.2 (19.5)	67.3 (24.7)	50.1 (22.3)	47.6 (29.5)	< 0.001
Global quality of life	62.1 (14.7)	53.6 (14.7)	38.1 (11.9)	33.3 (9.6)	< 0.001
Symptoms***					
Fatigue	19.7 (19.7)	40.4 (20.9)	61.9 (16.6)	68.2 (11.8)	< 0.001
Nausea and vomiting	11.1 (19.7)	4.3 (9.9)	10.3 (16.2)	9.5 (16.2)	0.17
Pain	29.1 (17.9)	45.3 (19.3)	68.2 (20.3)	83.3 (13.6)	< 0.001
Dyspnea	15.8 (21.7)	28 (25.5)	42.8 (12.5)	61.9 (12.5)	< 0.001
Sleep difficulties	9.1 (20.3)	20.6 (25.9)	33.3 (31.6)	42.8 (16.2)	< 0.001
Appetite loss	18.1 (25.1)	20 (24.2)	38.1 (28.4)	57.1 (16.2)	< 0.001
Constipation	33.8 (30.6)	30 (29.5)	34.9 (32.4)	19.1 (17.8)	0.58
Diarrhea	23.1 (28.5)	20 (26.1)	17.4 (27.1)	9.5 (16.2)	0.57
Financial difficulties	32.2 (25.4)	52.6 (23.4)	57.1 (23.9)	66.6 (27.2)	< 0.001

* Derived from Kurskal-Wallis test; ** Higher scores indicate better conditions; *** Higher scores indicate worse conditions.

**Table 3:** Item-scale Correlation Matrix for Azeri Version of EORTC QLQ-C30*

	PF	RF	EF	CF	SF	GQOL	FA	N&V	PA
Physical functioning (PF)									
Q1	0.856**	0.622**	0.476**	0.529**	0.400**	0.535**	-0.636**	-0.091	-0.607**
Q2	0.855**	0.528**	0.355**	0.509**	0.417**	0.525**	-0.572**	-0.118	-0.592**
Q3	0.791**	0.528**	0.386**	0.540**	0.289**	0.348**	-0.472**	-0.083	-0.456**
Q4	0.874**	0.525**	0.432**	0.626**	0.406**	0.506**	-0.594**	-0.019	-0.595**
Q5	0.824**	0.595**	0.496**	0.526**	0.385**	0.518**	-0.572**	-0.062	-0.605**
Role functioning (RF)									
Q6	0.561**	0.933**	0.474**	0.434**	0.498**	0.555**	-0.593**	-0.056	-0.611**
Q7	0.681**	0.936**	0.438**	0.477**	0.479**	0.564**	-0.597**	-0.050	-0.643**
Emotional functioning (EF)									
Q21	0.372**	0.346**	0.782**	0.473**	0.230**	0.372**	-0.457**	-0.161	-0.499**
Q22	0.394**	0.328**	0.837**	0.406**	0.200**	0.464**	-0.474**	-0.219*	-0.573**
Q23	0.315**	0.333**	0.823**	0.303**	0.252**	0.362**	-0.463**	-0.188*	-0.457**
Q24	0.535**	0.542**	0.735**	0.405**	0.462**	0.464**	-0.505**	-0.119	-0.476**
Cognitive functioning (CF)									
Q20	0.643**	0.463**	0.554**	0.915**	0.434**	0.472**	-0.443**	-0.162	-0.531**
Q25	0.484**	0.392**	0.296**	0.849**	0.387**	0.305**	-0.380**	-0.049	0.373**
Social functioning (SF)									
Q26	0.390**	0.344**	0.224**	0.429**	0.870**	0.432**	-0.335**	-0.043	-0.318**
Q27	0.414**	0.569**	0.404**	0.401**	0.902**	0.578**	-0.496**	-0.037	-0.509**
Global quality of life (GQOL)									
Q29	0.595**	0.586**	0.547**	0.425**	0.529**	0.974**	-0.642**	-0.010	-0.731**
Q30	0.544**	0.582**	0.477**	0.454**	0.590**	0.978**	-0.608**	-0.011	-0.668**
Q10	-0.658**	-0.602**	-0.489**	-0.399**	-0.419**	-0.572**	0.830**	0.093	0.665**
Q12	-0.522**	-0.491**	-0.491**	-0.396**	-0.378**	-0.480**	0.855**	0.053	0.581**
Q18	-0.537**	-0.517**	-0.517**	-0.392**	-0.403**	-0.569**	0.847**	0.186*	0.706**
Nausea and Vomiting (N&V)									
Q14	0.030	-0.027**	-0.027**	-0.119**	-0.013**	-0.028	0.038	0.950**	0.107**
Q15	0.004	-0.088**	-0.088**	-0.120**	-0.080**	-0.038	0.028	0.913**	0.177**
Pain (PA)									
Q9	-0.555**	-0.541**	-0.541**	-0.394**	-0.351**	-0.601**	0.610**	0.246**	0.871**
Q19	-0.632**	-0.620**	-0.620**	-0.512**	-0.472**	-0.645**	0.730**	0.009	0.871**

* Positive correlation for functioning subscales and negative correlation for symptom subscales; ** All are at < 0.01 significant levels.

**Table 4:** Cronbach's alpha Coefficient for the Azeri Version EORTC QLQ-C30

	Item	Mean (SD)	Cronbach’s alpha
Functioning**			
Physical	5	82.7 (20.6)	0.89
Role	2	75.3 (21.6)	0.85
Emotional	4	66.1 (21.4)	0.80
Cognitive	2	80.2 (25.1)	0.71
Social	2	70.1 (25.4)	0.73
Global quality of life	2	54.2 (17.1)	0.94
Symptoms***			
Fatigue	3	36.2 (25.5)	0.79
Nausea and vomiting	2	8.3 (16.1)	0.83
Pain	2	43.7 (24.6)	0.68
Dyspnea	1	26.7 (26.7)	-
Sleep difficulties	1	18.7 (26.1)	-
Appetite loss	1	23.8 (26.7)	-
Constipation	1	31.8 (29.94)	-
Diarrhea	1	20.4 (26.8)	-
Financial difficulties	1	45.2 (27.1)	-

## Discussion

This study reported on translation and validation of the EORTC QLQ-C30 in Azerbaijan and proved that the Azeri version of the questionnaire is valid. With one exception (Camran et al., 2018) we could not identify any other studies that report on psychometric properties of patient-reported outcomes from Azerbaijan. Thus one should bear in mind that such studies in Azerbaijan are at their early stages. Perhaps with introducing this questionnaire for measuring health related quality of life in cancer patients we could see more publications from Azerbaijan on this topic in the future.

This study took a very straightforward procedure in translating and validating the EORTC QLQ-C30 in Azerbaijan for use as a standard and valid benchmark in clinical and epidemiological studies. In general patients did not report any serious problems while completing the questionnaire and hopefully we could find all equivalent words in Azeri for the English expressions. Sometimes one major problem in translating well-known questionnaires into other languages of the origin is the fact that findings equivalents are very difficult (Kleijn et al., 2006).

Know-groups comparison was performed to assess discriminant validity. Almost in all measures significant differences were observed among patients with different stages of the disease. However, the result for three symptom subscales (nausea and vomiting, constipation and diarrhea) among patients who differed in disease stage was not significant. It seems that since the first line treatment for patients was surgery (surgical resection and flap reconstruction), thus these three symptoms (usually seen among patients who receive chemotherapy) did not show significant differences. In addition the findings showed a good item-scale correlation for all functioning and multi-item symptom subscales, which lend support to the hypothesized scale structure of the questionnaire.

The Azeri version of the EORTC QLQ-C30 showed good internal consistency where all Cronbach’s alpha coefficients exceeded the expected threshold value (alpha ≥ 0.7). However the Cronbach’s alpha coefficient for pain subscale was 0.68, which seems acceptable. The finding from our study was almost similar to other studies carried out in different countries (Kobayashi et al., 1998; Montazeri et al., 1999; Zhao and Kanda, 2000; Arraras et al., 2002; Guzelant et al., 2004; Awad et al., 2008; Wan et al., 2008).

We studied a cohort of oral cancer patients. The descriptive findings indicated that overall the score for global quality of life subscale was the lowest and score for the physical functioning was the highest. Perhaps this indicates that although patients who diagnosed with oral cancer might have good physical functioning or physical health, they suffer from low global quality of life and emotional functioning as reflected by patients. Also patients relatively scored higher on pain subscales compared to other symptom subscales, which confirms such observation. There is evidence that cosmetic appearance, and psychological well-being can become compromise during the diagnosis, treatment, and survivorship of patients with oral cancer (Valdez and Brennan, 2018).

This was a preliminary validation study and surely might have some limitations. Limited psychometric evaluations, studying only oral cancer patients, and lack of follow-up information are among important shortcomings with this study. However, we are very optimistic that such studies could introduce patient-reported outcomes to the Azeri medical professionals and might help clinicians and medical investigators to consider patients’ voice and concerns. The EORTC QLQ-C30 has been approved for clinical use and scientific interpretation in numerous studies and has proven to be an important link between patient and physician.

In conclusion, the findings from this preliminary validation study indicated that the Azeri version of the EORTC QLQ-C30 is a valid core instrument for measuring quality of life in cancer patients in Azerbaijan.

## Data Availability

All data presented in this paper are available from the corresponding authors on a reasonable request.

## Abbreviations

EORTC:

QLQ-C30

## References

[B1] Aaronson NK, Ahmedzai S, Bergman B (1993). The European Organization for Research and Treatment of Cancer QLQ-C30: a quality-of-life instrument for use in international clinical trials in oncology. J Nat Cancer Institut.

[B2] Arraras JI, Arias F, Tejedor M (2002). The EORTC QLQ-C30 (version 30) quality of life questionnaire: validation study for Spain with head and neck cancer patients. Psychooncology.

[B3] Awad MA, Denic S, El Taji H (2008). Validation of the European Organization for Research and Treatment of Cancer Quality of Life Questionnaires for Arabic-speaking populations. Ann N Y Acad Sci.

[B5] Davudov MM, Harirchi I, Arabkheradmand A (2019). Evaluation of quality of life in patients with oral cancer after mandibular resection: Comparing no reconstruction, reconstruction with plate, and reconstruction with flap. Medicine.

[B7] Gegechkori N, Haines L, Lin JJ (2017). Long term and latent side effects of specific cancer types. Med Clin North Am.

[B8] Guzelant A, Goksel T, Ozkok S (2004). The European Organization for Research and Treatment of Cancer QLQ-C30: an examination into the cultural validity and reliability of the Turkish version of the EORTC QLQ-C30. Eur J Cancer Care.

[B9] Jehn P, Stier R, Tavassol F (2019). Physical and psychological impairments associated with mucositis after oral cancer treatment and their impact on quality of life. Oncol Res Treat.

[B10] Jitender S, Mahajan R, Rathore V (2018). Quality of life of cancer patients. J Exp Ther Oncol.

[B11] Kamran S, Sharif M, Garay G (2019). Validation of the Azeri version of the pediatric epilepsy side effects questionnaire. Childs Nerv Sys.

[B12] Kleijn WC, Ogoshi K, Yamaoka K et al (2006). Conceptual equivalence and health-related quality of life: an exploratory study in Japanese and Dutch cancer patients. Qual Life Res.

[B13] Kobayashi K, Takeda F, Teramukai S (1998). A cross-validation of the European Organization for Research and Treatment of Cancer QLQ-C30 (EORTC QLQ-C30) for Japanese with lung cancer. Eur J Cancer.

[B14] Montazeri A, Harirchi I, Vahdani M (1999). The European Organization for Research and Treatment of Cancer Quality of Life Questionnaire (EORTC QLQ-C30): translation and validation study of the Iranian version. Supportive Care Cancer.

[B15] Naughton MJ, Weaver KE (2014). Physical and mental health among cancer survivors: considerations for long-term care and quality of life. N C Med J.

[B16] Shapiro CL (2018). Cancer Survivorship. N Engl J Med.

[B18] Valdez JA, Brennan MT (2018). Impact of oral cancer on quality of life. Dent Clin North Am.

[B19] Wan C, Meng Q, Yang Z (2008). Validation of the simplified Chinese version of EORTC QLQ-C30 from the measurements of five types of inpatients with cancer. Ann Oncol.

[B20] Yun YH, Park YS, Lee ES (2004). Validation of the Korean version of the EORTC QLQ-C30. Qual Life Res.

[B21] Zhao H, Kanda K (2000). Translation and validation of the standard Chinese version of the EORTC QLQ-C30. Qual Life Res.

